# 634. Incidence of Firepit and Fireplace Related Burn Injuries in the United States

**DOI:** 10.1093/jbcr/irag033.460

**Published:** 2026-04-06

**Authors:** Rajan K Thakkar, Nathaniel Klingele, Rebecca J McAdams, Dana M Schwartz, Renata Fabia, Lara B McKenzie

**Affiliations:** Nationwide Children's Hospital, Columbus, OH; Nationwide Children's Hospital, New Albany, OH; Nationwide Children's Hospital, Columbus, OH; Nationwide Children's Hospital, Columbus, OH; Nationwide Children's Hospital, Columbus, OH; Nationwide Children's Hospital, Columbus, OH

## Abstract

**Introduction:**

Burn injuries from firepits, fireplaces, and related equipment are a common reason for emergency department visits. Burns can affect people of all ages, but the pediatric population may be at a heightened risk due to their developmental stage and behaviors. Despite popularity among heating and recreational devices, national data describing how and where these injuries occur are limited. Understanding the incidence and patterns of firepit and fireplace related burn injuries can help guide prevention strategies and improve safety.

**Methods:**

We conducted a retrospective analysis by using the National Electronic Injury Surveillance System data for patients of all ages who presented to a US emergency department (ED) for a burn-related injury from firepits, fireplaces, or related equipment from 2012-2024. Odds ratios adjusted for sex with corresponding 95% confidence intervals (CIs) were generated with multivariate logistic regression models. Injury rates per 100 000 population were calculated by using US Census Bureau data. Trends in these rates were assessed using Joinpoint regression software. We generated national estimates from 1641 cases.

**Results:**

An estimated 56 340 burn injuries associated with firepits, fireplaces, and equipment (95% CI: 43854-68 826) were treated in US EDs from 2012-2024, equating to about 12 per day. Injured patients were most often males (65%), burned their hands (45%), and had contact (87%) with a firepit, fireplace, or related equipment. Patients fell into the fire 40% of the time. The rate of burns remained steady throughout the study period, average as 1.3 per 100 000 population. Half (50%) of those injured were ≥ 16 years and 34% were children ≤4 years. The rate of burn injury for children ≤4 was 7.8 times that of those ≥16 years. Most often, patients ≥16 years were burned by contacting a firepit, while children ≤4 years were burned by contacting the glass or screen of a fireplace. Nearly one-fifth (17%) of injuries resulted in hospitalization. Children ≤4 years had four times the odds of a hand burn compared to patients of all other ages (aOR = 4.09, 95% CI: 2.83-5.90).

**Conclusions:**

Burn injuries from firepits and fireplaces remain a significant health concern, with children, particularly those ≤4 years old, experiencing the highest rates most often involving the hands. These injuries can lead to long term scarring and functional impairment, highlighting the need for pediatric-focused intervention through safer product design and caregiver education. Continued attention to prevention across all age groups is essential to reduce emergency department visits and hospitalizations related to these common household hazards.

**Applicability of Research to Practice:**

The incidence of fireplace and firepit related injuries remain high and leads to significant rates of hospitalization in all ages.

**Funding for the study:**

N/A.

